# Estimation of Power Output and Efficiency of Induction Motors: A New Non-Intrusive Approach

**DOI:** 10.3390/s25030754

**Published:** 2025-01-26

**Authors:** Paula Paramo-Balsa, Juan Manuel Roldan-Fernandez, Jorge Semião, Manuel Burgos-Payan

**Affiliations:** 1 Department of Electrical Engineering, Universidad de Sevilla, 41092 Sevilla, Spain; pparamo@us.es; 2 Departamento de Engenharia Electrotécnica, Instituto Superior de Engenharia, Universidade do Algarve, 8005-139 Faro, Portugal; jsemiao@ualg.pt

**Keywords:** induction motors, non-intrusive methods, power estimation, efficiency estimation, energy management systems, industry 4.0

## Abstract

Industry 4.0 (I4.0) represents a transformative approach, integrating technology, production methods, and information and communication technology to enhance industrial value creation. A central I4.0 goal in the energy domain is improving energy efficiency to boost industrial competitiveness and profitability. Given that induction motors account for nearly two-thirds of industrial electrical energy consumption, optimizing their efficiency is crucial. Energy management systems (EMSs) need real-time data to assess motor efficiency, enabling prompt identification and replacement of inefficient motors with alternatives that have optimal efficiency class and rated power for specific applications. This paper introduces a novel non-intrusive method for estimating the load and efficiency of induction motors without disrupting their operation. To reach that goal, the proposed method optimizes the parameters of a set of relationships between output power, input power, and losses with the motor speed, minimizing the error in the estimates. It requires only input electrical power and motor speed measurements to set the model parameters and estimates the load and efficiency using either speed or input power measurements. The experimental results demonstrate that the proposed method, with a mean overall error of less than 3.5% in estimating output power and efficiency, outperforms conventional methods.

## 1. Introduction

Industry 4.0 (I4.0), the industrial revolution currently underway, also known as smart manufacturing or simply digitalization, refers to the process of combining technologies, production methods, and information and communication technology to increase the value creation of the industry [1]. One of the main objectives of the I4.0 approach, particularly regarding energy, is to improve the efficiency of the industry, producing the same products with less energy. This, in turn, reduces the cost of electricity bills, which increases the competitiveness and profits of companies.

Induction motors, as the main suppliers of mechanical power for industry [2], account for about 68% of the world’s electrical energy consumption in industry [3], and nearly 90% of electric drives are powered by squirrel cage induction motors [4,5].

Energy policy institutions have long recognized that improving the efficiency of three-phase induction motors is a technically feasible and economically viable strategy to simultaneously reduce industry energy demand and greenhouse gas emissions. Examples of such policies include Minimum Efficiency Performance Standard (MEPS) regulations and Motor Efficiency Classes (MECs). However, using a motor with a high international efficiency classification does not guarantee the machine’s efficiency in service since motors rarely operate at full load continuously. In the industrial environment, motors operate, on average, at 60% of their full load [6]. On the other hand, the ratio between the actual mechanical power needed by the driven mechanical load and the rated power of the motor, i.e., the load ratio, together with its variation over time, also plays a key role in achieving the desired energy efficiency.

As can be seen, identifying motors that operate inefficiently is a primary task to meet both I4.0 and institutional energy objectives [7]. This will be the first step in a series of informed decisions enabling the replacement of these induction motors with others whose rated powers and efficiency classes are better suited to the required service conditions. So, to achieve the goal of improved efficiency, energy management systems (EMSs) need real-time information on the efficiency of a plant’s induction motors to monitor their performance. Those real data logs will be the basis for making informed decisions about whether to replace or maintain them.

Given the vast number of motors in industry, estimated at approximately 2230 million units worldwide [8], there has been a long-standing interest in different methods for monitoring their load and efficiency without interfering with their operation. Only with this knowledge can we analyze the actual energy and economic performance of the motors (production costs) and make informed decisions about whether to keep or replace them.

Although there is no standardized method for classifying the degree of intrusiveness of a test or measurement, it is possible to make a qualitative distinction between intrusive and non-intrusive tests or measurements. An intrusive test or measurement, in the most extreme cases, would need to shut down the induction motor and, consequently, the production line it supports. In contrast, a non-intrusive test or measurement, at its most extreme, could be conducted without any disruption to the motor’s operation [6].

Tsybikov et al. present a comparison of the main international standards used to determine the efficiency of direct-on-line (DOL) three-phase induction motors from experimental tests [9], including IEC 60034-2 [10] and IEEE Standard 112 [11]. Unfortunately, the methods specified in these standards require a torque meter or dynamometer, as well as measurements of winding resistance and temperature. They also require an extensive set of tests, both under variable load and no-load conditions, with a variable voltage power supply, which is impractical under field conditions.

As a result of the absence of a standardized procedure for determining the load and efficiency of a motor in service, numerous studies have been developed to estimate these quantities [6,12]. Sousa-Santos et al. [13], Geravandi and CheshmehBeigi [14], and Hsu et al. [15] provide an extensive review of the methods for estimating the efficiency of induction motors. The nameplate, slip, and the current methods are highlighted for their low level of intrusiveness, making them widely used in industry. However, researchers like Dlamini et al. note that estimates from these methods are negatively affected by their dependence on nameplate data, as standards allow wide tolerance values for these data [16].

The method of the current is based on the simplifying assumption that the output power of the motor is proportional to the current [6]. While simple to use, this method lacks precision, so it is advisable not to use it for light loads less than 50% [16]. In the air-gap torque method, as analyzed by Stopa et al. [17] and Salomon et al. [18], among others, the additional load losses are assigned from typical values expressed as percentages of the rated power output, depending on the machine size, as defined in IEEE Standard 112 [11]. This method requires the measurement or estimation of the stator resistance and the friction and windage power losses based on the motor’s rated power. However, the method overlooks iron power losses, which contributes to increased estimation errors. Geravandi and Moradi [19] also present an efficiency estimation technique based on the air-gap torque method and the simulation of 425 induction motors in MATLAB software (version: R2022b). They use the dispersion of the test data and non-linear regression to calculate the windage and friction losses as well as the rotor stray load losses. The estimation of the motor speed and the stator resistance is also required, for which the voltages, currents, and nameplate information are used. All these parameters’ estimations and the use of nameplate information increase the error of the efficiency estimation.

**Figure 1:**
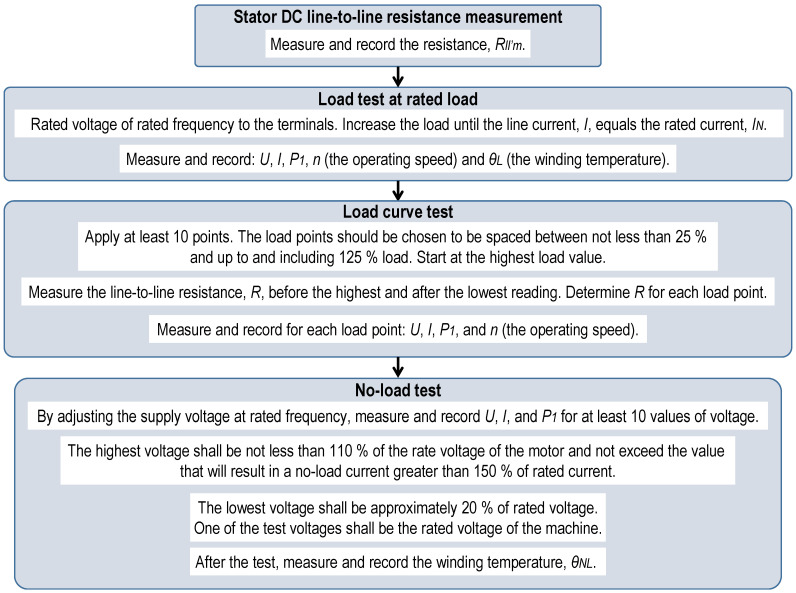
Diagram of the procedure for determining a motor’s circuit parameters according to IEC 60034-28:2013.

**Figure 2:**
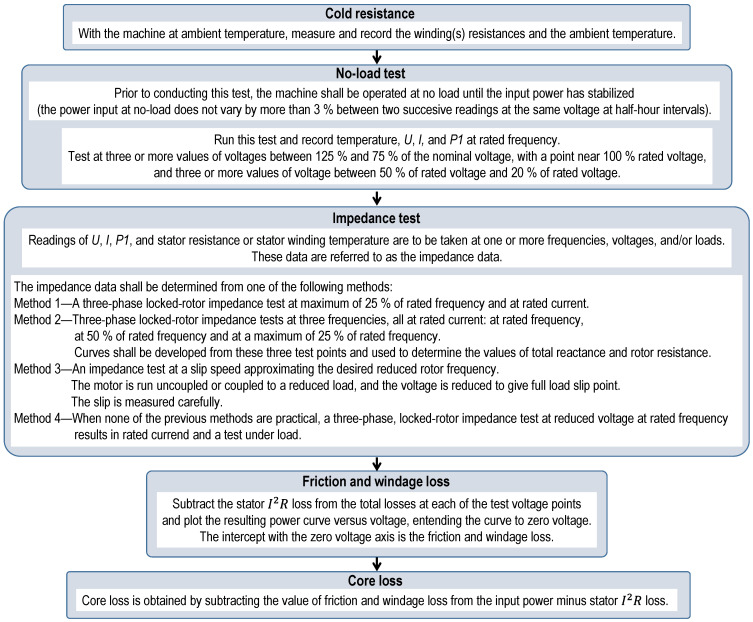
Diagram of the procedure for determining a motor’s circuit parameters according to IEEE Standard 112 Method F/F1.

The equivalent circuit method requires a comprehensive set of tests, which is challenging to conduct under field conditions [20]. For instance, the standard procedures for determining the parameters of a motor’s circuit model are outlined in IEC 60034-28:2013 [21] and IEEE Standard 112 Method F/F1 [11]. The procedures of IEC 60034-28:2013 are illustrated in Figure 1, while those of IEEE Standard 112 Method F/F1 are sketched in Figure 2.

Other authors, such as Lu et al. propose an equivalent circuit for the motor using terminal quantities and nameplate data [22]. They replace the no-load test with empirical information, and the additional load losses are estimated according to IEEE Standard 112. El-Ibiary suggests a method to estimate efficiency using voltage, current, electrical power, stator resistance, temperature, and motor speed [23]. The model parameters are obtained from variables measured at no load and at one or two operating points. Consequently, the equivalent circuit method is impractical for a motor in service since it requires tests that are difficult to perform under field conditions [15].

There are other methods that use the idea of loss summation/separation, such as IEC 60034-2-1 [10] and IEEE Standard 112 [11]. To this end, authors such as Gajjar et al. propose adaptations to field conditions [24]. However, all these approaches require the incorporation of additional simplifications, or heuristic information concerning certain components of power losses. These substitution options are needed to compensate for information not available because the necessary tests to obtain it cannot be performed under field conditions. For instance, Esen and Özdemir suggest a simplification of the IEC tests to determine motors’ efficiency, adjusting the results with coefficients derived from information from 86 tests conducted on 48 different induction motors [8]. According to IEC 60034-2-1 [10], iron losses and friction and windage losses are calculated from the no-load test at different voltage levels [10]. However, the method proposed in [8] requires only one no-load measurement point at rated voltage. To assess the efficiency using the proposed method, both the load test (if possible) and the no-load test should be conducted, and the nameplate information should be used. Pillay et al., based on an extensive set of tests with 182 induction motors, propose a different expression from that in IEEE Standard 112 to determine additional load losses [25]. Something similar is proposed by Al-Badri and Pillay [26]. Consequently, the loss segregation-based method and its variants are not well-suited for in-service motors due to their intrusive nature and the requirement for heuristic generic information and/or estimations from other motors, which negatively impact their accuracy.

Other methods use computer tools to estimate the efficiency of induction motors [6]. These methods rely on different software packages developed from databases containing information from many motors. However, these methods are not suitable for field conditions, mainly due to test requirements. They also require generic information from a private database to estimate the efficiency of the motors.

Optimization methods are also used to estimate motor efficiency. These methods are based on heuristic techniques and evolutionary algorithms (HTEAs) that allow for the estimation of equivalent circuit parameters and quantities, such as motor efficiency. For example, Ghasemi-Bijan et al. use genetic algorithms to estimate the parameters of the equivalent circuit, thereby obtaining the motor’s efficiency [27]. The method requires data from the nameplate and information from a database of 129 motors. Something similar is proposed in [28]. In general terms, these methods are not suitable for use in field conditions, due to their high intrusiveness and the need to replace some motor-specific information with average data taken from private motor databases [29], which negatively affects the estimates.

Our literature review shows that there is no non-invasive procedure available for determining the power output and efficiency of a motor under field or service conditions. Standardized procedures for determining output power and efficiency require measuring the output torque (torque meter) and evaluating the mechanical output power from a comprehensive set of tests that are only feasible to perform under well-equipped test facility conditions.

For a motor in service, the current state of the art only offers the possibility of an indirect estimation of the mechanical load and efficiency. This often requires information from the nameplate, the measurements of the stator resistance, and winding temperature, along with some approximations and/or heuristic generic information from other motors. This nonspecific information, which replaces unavailable actual motor data, is generally not free or publicly available and ultimately deteriorates the expected accuracy of the estimations.

To address this gap, this work proposes a new, non-intrusive, and low-cost method for estimating the mechanical power output and efficiency of a DOL motor in service. It requires only the measurements of the electrical power input and the rotor’s speed to set the parameters of the model. Once the estimation model has been identified, it can be fed with speed or, better, with input power measurements to estimate the power output and efficiency. The method does not require any nameplate information, measurements of the stator winding resistance or its temperature, heuristic information, or data from catalogs or databases. This makes the proposed method robust, even against variations in the motor’s own parameters (or working conditions), due to aging, repairs, and/or refurbishments. The proposed method is based on the following:

- An approximation of the power–speed relationship, to which an approximation of the total losses power is added, similar to the proposal in IEC/TS 60034-31 [30] and used in [31] for evaluating the energy consumption and life cycle costs of a motor. This allows, in turn, for an approximation of the electrical power input based on speed.
- The optimization of the values of the coefficients involved in the approximation of the input power, in a way that minimizes errors in the estimations.

The required measurements, electric power input, and speed can be easily accessed from the Supervisory Control and Data Acquisition (SCADA) or Motor Control Center (MCC). In the case that motor speed measurements were not available in the SCADA or MCC, they could be easily integrated with almost no intrusion [32,33], making the proposed method suitable for use in field conditions. In the less frequent case that the electrical power input of the motor was not monitored, it would be necessary to install a simple low-cost wattmeter on the motor power line. Of course, it will also be necessary to integrate the wattmeter information into the SCADA system, but this is a routine task that must be completed to integrate the information from any other device of the plant. Moreover, since information regarding electrical power and speed can be obtained online, or almost continuously, the volume of data that can be collected to identify the motor could be enormous. This suggests a way to improve the optimization process, which could lead to a more accurate identification of the motor model.

The main contributions of this work are as follows:

- This work proposes a new and different way of approaching the problem of online non-intrusive estimation of the load and efficiency of induction motors. The proposed method is based on approximating the relationships between output power and speed, and between power losses and output power, requiring only measurements of the input electrical power and motor speed (SCADA) to set the model parameters, and only the speed or the input power to estimate the load and efficiency.
- The experimental results show that the proposed method outperforms conventional methods used in industry.
- The estimates improve as the number of measurements used in the identification stage increases, which is a very relevant and distinctive characteristic of the proposed method.
- The proposed method is very well-suited for field or in-service conditions, as it only requires readily accessible measurements, such as the input electrical power and speed (SCADA) in the identification stage, and only one of them (input power or speed) in the estimation stage.
- The proposed method also does not require information on any motor-specific parameters or nameplate data, allowing the method to be applied to motors that are even aged, repaired, rewound, or refurbished, distinguishing it from conventional methods and highlighting its potential as a practical tool that could help industrial plants achieve efficiency goals.

Following this introduction, the remainder of this paper starts in Section 2 by reviewing the work performed to estimate the efficiency and output power of induction motors used in industry. It also provides the basic foundations behind each of the main conventional methods described in the state of the art. Then, the proposed method is introduced, and the experimental testbed is described in Section 3. Next, in Section 4, the results obtained in this study are presented and compared with the experimental outcomes and with the conventional estimation methods used in industry. A short discussion is also included to close this section. Finally, in Section 5, the main findings and conclusions are drawn and summarized.

## 2. Load and Efficiency Estimation Methods

The efficiency of a motor, *η*, is defined as the ratio of the mechanical output power, *P*_2_, to the electrical input power, *P*_1_:

(1) $$\eta =\frac{P_{2}}{P_{1}}$$

However, motors rarely operate steadily under a constant load. As the motor load often varies over time during its duty cycle, the efficiency of the motor also varies. To incorporate the effect of load variations in the work cycle, it is necessary to include the effect of time, transitioning from power to energy. If *P_2_*(*t*) and *P*_1_(*t*) represent the variations in output and input powers during the motor’s duty cycle period (a year, for example), *t_T_*, the quantities of input (electrical) energy, *E*_1_, and output (mechanical) energy, *E*_2_, can be expressed as:

(2) $$E_{1}=\int_{0}^{t_{T}}P_{1}\left(t\right)\cdot dt=\int_{0}^{t_{T}}\frac{P_{2}(t)}{\eta \left(t\right)}\cdot dt=\int_{0}^{t_{T}}(P_{2}\left(t\right)+P_{L}\left(t\right))dt$$

(3) $$E_{2}=\int_{0}^{t_{T}}P_{2}\left(t\right)\cdot dt=\int_{0}^{t_{T}}\eta \left(t\right)\cdot P_{1}\left(t\right)\cdot dt=\int_{0}^{t_{T}}(P_{1}\left(t\right)-P_{L}\left(t\right))dt$$

where *P_L_*(*t*) are the total power losses. Consequently, the energy use efficiency, or the motor’s energy efficiency over its duty cycle, *η_E_*, can be defined as:

(4) $$\eta_{E}=\frac{E_{2}}{E_{1}}=\frac{\int_{0}^{t_{T}}P_{2}\left(t\right)\cdot dt}{\int_{0}^{t_{T}}P_{1}\left(t\right)\cdot dt}=1-\frac{\int_{0}^{t_{T}}P_{L}\left(t\right)\cdot dt}{\int_{0}^{t_{T}}P_{1}\left(t\right)\cdot dt}$$

As can be seen, to determine the energy efficiency of a motor operating with a specific duty cycle, it is necessary to have the following:

- The evolution of the electrical input power, *P*_1_(*t*), during its duty cycle, *t* ∈ (0, *t_T_*), which is easily accessible and can be measured/recorded with no (or minimal) intrusion;
- The evolution of the output power, *P*_2_(*t*), or alternatively, the power losses, *P_L_*(*t*), during its duty cycle *t* ∈ (0, *t_T_*). None of these quantities are easily accessible or measurable.

Therefore, the challenge when evaluating the efficiency, or rather, the energy efficiency of industrial motors, lies mainly in the difficulty of measuring the mechanical power output, *P*_2_, and its variation over time. This process is costly with current technology and extremely complicated (practically unfeasible) under field conditions. This highlights the merit of methods for estimating the mechanical load and efficiency of in-service induction motors.

Ferreira and Almeida [16] and Sousa-Santos et al. [13] evaluated the most common in-field induction motor load estimation methods used in industry. They focused on the methods that require less complexity in terms of measurement equipment and data processing, as well as those that offer better expected accuracy. Accordingly, methods involving the equivalent circuit, segregated losses, computer tools, and optimization methods based on HTEA, were all excluded from the assessment. According to Sousa-Santos et al. [13] and Ferreira and Almeida [16], this work will consider the most common methods for estimating load and efficiency of the in-field induction motors used in industry:

- Nameplate;
- Slip;
- Current;
- Air-gap torque.

### 2.1. Nameplate Method

This method estimates the output power using a measurement of the input power and the motor’s nameplate information, thus presenting a low level of intrusion [13]. In this method, it is assumed that the motor’s efficiency, *η*, is constant and equal to the rated value, *η_N_*, regardless of the motor’s load condition. From the measurement of the motor’s input power, *P*_1_, and the full-load efficiency value (nameplate), *η_N_*, the output power is estimated as

(5) $$P_{2}=P_{N}\cdot \frac{\eta \cdot P_{1}}{\eta_{N}\cdot P_{1N}}≃P_{N}\cdot \frac{P_{1}}{P_{1N}}=\eta_{N}\cdot P_{1}$$

where *P*_1*N*_ is the full-load input power. The method lacks precision for low-load conditions due to variations in total power losses and efficiency across different loads. Moreover, the values from the nameplate are required, which are quantities subject to acceptable tolerances by standards that, in some cases, are quite significant, as shown in Table 1 [34].

For example, for a motor with a power of less than 150 kW, if the rated efficiency listed on the nameplate is 0.75 p.u., it can actually have an efficiency ranging between that value, 0.75 p.u., and 0.75 − 0.15·(1 − 0.75) = 0.71 p.u. This means a 4% admissible tolerance in the full-load efficiency. Another practical difficulty of the method is that, sometimes, the nameplate information is not always available (due to lost or painted-over plates), or it may not be reliable, because the motor has undergone repairs (bearings) and rewinding, so the nameplate values cannot be guaranteed.

**Figure 3:**
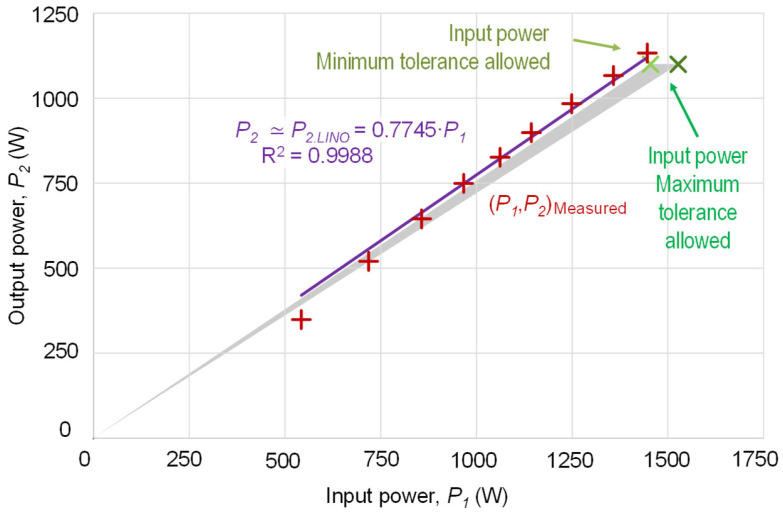
Relationship between the input power of the induction motor, *P*_1_, and the output power, *P*_2_, with measured values. Regression fitting line passing through the origin and the gray triangle of indeterminacy due to tolerances.

In Figure 3, a set of points (*P*_1_, *P*_2_) is shown with the measurements of the input electrical power, *P*_1_, and the output power, *P*_2_, corresponding to a 1.1 kW motor powered at rated voltage.

By requiring the regression line to include the origin of coordinates, the fitting line, *P_2,LIN_*_0_, only needs one coefficient:

(6) $$P_{2}(P_{1})≃P_{2,LIN0}(P_{1})=0.7745\cdot P_{1}\;R^{2}=0.9988$$

Since measurements of the output power, *P*_2_, will not be available, the coefficient from (6) cannot be calculated by regression. This requires calculating the coefficient, as in (5), using the motor nameplate rated efficiency.

This highlights the importance of the precision of the point selected or considered as the rated point (nameplate tolerance). Figure 3 also illustrates the triangle of indetermination, the set of lines, *P*_2*LIN*0_ ≈ (*P_N_*/*P*_1*N*_)·*P*_1_ = *η_N_*·*P*_1_, assignable to the permissible tolerances for the electrical input power at full load or to the rated efficiency. In this case, it can be observed that with mechanical loads near full load, the output power tends to be overestimated, whereas with lower mechanical loads, the output is underestimated.

### 2.2. Slip Method

This method estimates the power output based on the motor’s rated speed (nameplate) and the speed measurement [6]. In its simplest form, it is considered that the relative or normalized power output is approximately equal to the ratio between the measured slip and the rated slip (nameplate) of the motor [13].

The mechanical power output, *P*_2_, is calculated from the motor’s rated power, *P_N_*, the rated slip, *s_N_*, and the value of the slip, *s*, as:

(7) $$P_{2}(s)=P_{N}\cdot \frac{P_{m}-P_{fw}-P_{LL}}{P_{mN}-P_{fwN}-P_{LLN}}≃P_{N}\cdot \frac{P_{m}}{P_{mN}}≃P_{N}\cdot \frac{U^{2}\cdot s\cdot \left(1-s\right)}{{U_{N}}^{2}\cdot s_{N}\cdot \left(1-s_{N}\right)}$$

where *P_m_* represents the gross mechanical power, *P_fw_* is the friction and windage losses, *P_LL_* is the additional load losses (stray-load losses), *P_mN_* is the gross mechanical power at full load, *P_fwN_* is the mechanical losses due to friction and ventilation at full load, *P_LLN_* is the additional losses at the rated load, *U* is the actual motor’s supply voltage, and *U_N_* is the rated voltage.

The main drawback of this method is that the load estimation is based on the value of the rated speed (slip) indicated on the nameplate, which has a tolerance acceptable by standards of up to 20%.

**Figure 4:**
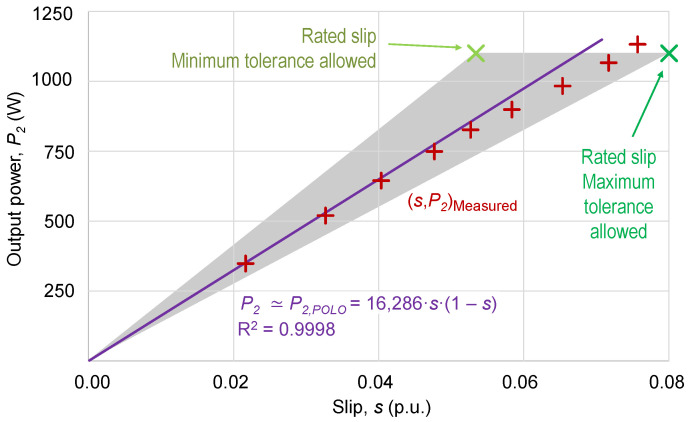
Relationship between slip, *s*, and output power, *P*_2_, with measured values and regression line passing through the origin. Gray triangle of indeterminacy due to tolerances.

Figure 4 shows the scatter plot of measured slip, *s*, and output power, *P*_2_, corresponding to a 1.1 kW motor, powered at rated voltage, including the indeterminacy triangle due to acceptable tolerances. By requiring the regression curve to include the origin of coordinates, the fitting second-degree polynomial, *P*_2,*POL*0_, only needs one coefficient:

(8) $$P_{2}\left(s\right)≃P_{2,POL0}\left(s\right)=16286\cdot s\cdot (1-s)\;R^{2}=0.9998$$

Since measurements of the output power, *P*_2_, will not be available, the coefficient in (8) must be calculated using the rated values of the output power and slip (nameplate), as in (7).

### 2.3. Current Method

This method uses the measurement of the current and the value of the nominal current (nameplate) to estimate the mechanical power output [6]. This method assumes that, regardless of the load, both the efficiency, *η*, and the power factor, *cosφ*, remain constant and equal to their nominal values. Thus, the mechanical power output is calculated as:

(9) $$P_{2}(I)=P_{N}\cdot \frac{{\eta \cdot P}_{1}}{\eta_{N}\cdot P_{1N}}{=P}_{N}\cdot \frac{\eta \cdot \sqrt{3}\cdot U\cdot I\cdot cos\varphi }{\eta_{N}\cdot \sqrt{3}\cdot U_{N}\cdot I_{N}\cdot cos\varphi_{N}}≃P_{N}\cdot \frac{U}{U_{N}}\cdot \frac{I}{I_{N}}$$

where *U_N_* is the motor’s rated voltage, *U* is the motor’s supply voltage, *I* is the measured current, and *η_N_*, *I_N_*, *cosφ_N_*, and *P_N_* are the rated values of efficiency, current, power factor, and output power, respectively.

However, the relation between the current and the output power (load) of a motor is not linear. This, along with the fact that both the efficiency and the power factor vary with the load, deteriorates the estimation.

**Figure 5:**
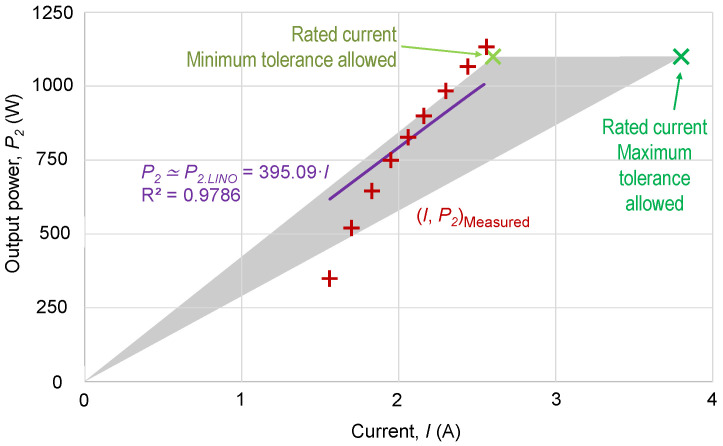
Relationship between motor current, *I*, and motor output power, *P*_2_, with measured values and regression line passing through the origin. Gray triangle of indeterminacy due to tolerances.

Figure 5 shows a set of points (*I*, *P*_2_) of motor current, *I*, and output power, *P*_2_, measured in a small 1.1 kW motor operating at rated voltage. By requiring the regression line to include the origin of coordinates, the regression line, *P*_2,*LIN*0_, has only one coefficient:

(10) $${P_{2}(I)≃P}_{2,LIN0}(I)=395.09\cdot I\;R^{2}=0.9786$$

In the absence of output power measurements, *P_2_*, the coefficient in (10) is derived from the nameplate, as outlined in (9).

Figure 5 also illustrates the triangle of uncertainty due to acceptable tolerances. In this case, it can be seen that near full load, the output power is underestimated, whereas with reduced loads, the output is overestimated.

### 2.4. Air-Gap Torque Method

This method aims to calculate the air-gap torque (power) from the motor’s input line voltages and currents, both instantaneous [35]. According to [36,37], if two of the line voltages, *v_AB_* and *v_CA_*, and the two line currents, *i_A_* and *i_C_*, are measured, the air-gap torque, *T_e_*, of a motor with *N_pp_* pairs of poles, is obtained as:

(11) $$T_{e}=\frac{N_{pp}}{\sqrt{3}}\{\left({2\cdot i}_{A}+i_{C}\right)\cdot \int_{0}^{t_{T}}\left[v_{CA}-R_{s}\cdot \left(i_{C}-i_{A}\right)\right]dt-\left(i_{C}-i_{A}\right)\cdot \int_{0}^{t_{T}}\left[v_{AB}-R_{s}\cdot \left(2\cdot i_{A}+i_{C}\right)\right]dt\}$$

where *t_T_* represents the duration of the steady state measurement record (30 s).

From that estimation of the air-gap torque, *T_e_*, the value of the power output, *P*_2_, could be obtained by deducting the additional load losses, *P_LL_*, and the friction and windage losses, *P_fw_*, from the gross mechanical power, *P_m_* = *T_e_*·*Ω*:

(12) $$P_{2}=P_{m}-P_{LL}-P_{fw}={\Omega \cdot T}_{e}-P_{LL}-P_{fw}$$

As seen in (11), in addition to the line voltages and currents, the value of the measurement of the stator resistance, *R_s_*, is required. It is worth noting that the stator voltage drop is due to the stator current, ***I_s_***, flowing through the stator impedance, ***Z_s_*** = *R_s_* + ***j***·*X_s_*, not only to the stator resistance. It is also important to note that (11) completely ignores the effect of the magnetic core power losses. On the other hand, since additional load losses, *P_LL_*, and friction and windage losses, *P_fw_*, cannot be measured in field conditions, they have to be estimated, typically as percentage values based on the rated power output (nameplate) of the motor according to IEEE Standard 112 [11].

**Figure 6:**
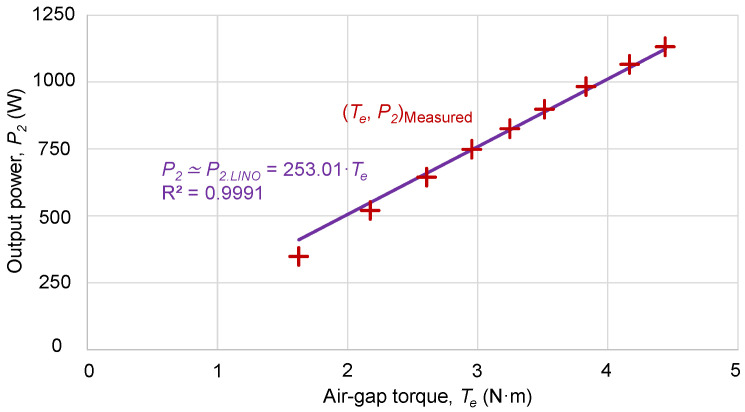
Relationship between the air-gap torque, *T_e_*, and the power output, *P*_2_, of the motor with measured values and regression line passing through the origin.

Figure 6 shows a set of points (*T_e_*, *P_2_*) of air-gap torque, *T_e_*, and output power, *P_2_*, measured in a small 1.1 kW motor, with a stator resistance *R_s_* = 2.3 Ω, powered at nominal voltage, and considering friction losses *P_fw0_* = 0.017·*P_N_* = 0.017·1100 = 18.7 W and additional load losses *P_LLN_* = 0.018·*P_N_* = 0.018·1100 = 19.8 W, according to IEEE Standard 112 [11].

By requiring the regression line to include the origin of coordinates, the regression line, *P*_2,*LIN*0_ (*T_e_*), has only one coefficient:

(13) $${P_{2}\left(T_{e}\right)≃P}_{2,LIN0}(T_{e})=253.01\cdot T_{e}\;R^{2}=0.9991$$

In the absence of the output power measurements, *P_2_*, the coefficient in (13) must be estimated from (11) and (12).

### 2.5. Summary of Conventional Methods

A survey of conventional methods shows that while the air-gap torque method is based on the power flow of the induction motor equivalent circuit model, the remaining methods (nameplate, slip, and current) are based on the proportionality between the mechanical power output and the electrical power input, the slip, or the current, respectively.

**Figure 7:**
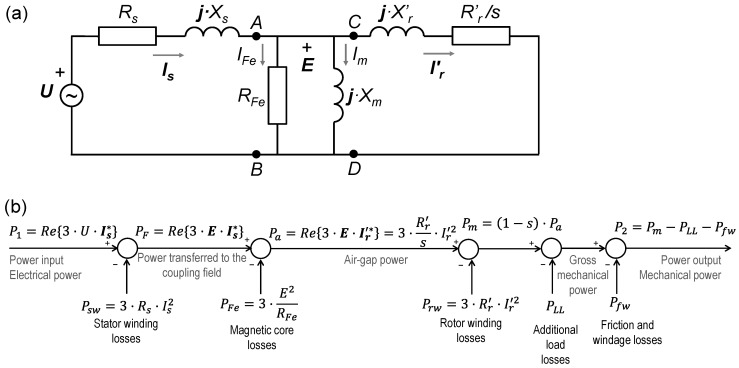
(**a**) T-type equivalent circuit model and (**b**) power flow and losses of an induction motor.

The top part of Figure 7 shows the T-type equivalent circuit model of an induction machine, based on IEC 60034-28 [21], with all the rotor quantities referring to the stator, while the bottom part illustrates the losses and power flow from the electrical power drawn from the mains to the mechanical power output.

From the equivalent circuit, the air-gap power, *P_a_*, corresponds to the power delivered to the equivalent rotor resistance, *R’_r_*/*s*, that is:

(14) $${P_{a}=\Omega }_{1}\cdot T_{e}=3\cdot \frac{R_{r}^{\prime }}{s}\cdot I_{r}^{\prime 2}=Re\left\{3\cdot E\cdot I_{r}^{\prime *}\right\}=Re\left\{3\cdot (U-Z_{s}\cdot I_{s})\cdot I_{r}^{\prime *}\right\}$$

where *U* is the supply voltage (phase angle reference), ***E*** is the phasor voltage in the magnetization transversal branch of the equivalent circuit, ***Z_s_*** = *R_s_* + ***j***·*X_s_* and ***I_s_*** are the stator impedance and current, respectively, ***I’_r_*** is the rotor current referring to the stator, and the symbol * is used to design the complex conjugate operator.

From the power flow, the air-gap power, *P_a_*, i.e., the active power input across terminals C-D in Figure 7, is the difference between the electrical power input, *P*_1_, and the sum of the stator winding losses, *P_sw_*, and the magnetic core losses, *P_fe_*. But the air-gap power can also be expressed as the quotient of the gross mechanical power, *P_m_*, and the difference one minus the slip, *s*, being the gross mechanical power, the sum of mechanical power output, the additional load losses, *P_LL_*, and the friction and windage losses, *P_fw_*:

(15) $${P_{a}=\Omega }_{1}\cdot T_{e}=P_{1}-P_{sw}-P_{fe}=\frac{P_{m}}{1-s}=\frac{P_{2}+P_{LL}+P_{fw}}{1-s}$$

Therefore, the calculation of the air-gap torque, *T_e_*, according to (11) is not actually based on the air-gap power, *P_a_* (the active power entering terminals C-D in Figure 7), but on the power transferred to the coupling field, *P_F_*, i.e., the active power entering terminals A-B in Figure 7. This power is the difference between the input power and the stator winding losses, *P_sw_*, or the sum of the air-gap power, *P_a_*, and the magnetic core losses, *P_fe_*. From the equivalent circuit, the power transferred to the coupling magnetic field, *P_F_*, corresponds to the active power input across terminals A-B in Figure 7:

(16) $$P_{f}=Re\left\{3\cdot E\cdot I_{s}^{*}\right\}=Re\left\{3\cdot (U-Z_{s}\cdot I_{s})\cdot I_{s}^{*}\right\}=P_{1}-P_{sw}=P_{a}+P_{fe}={Ω}_{1}\cdot T_{e}+P_{fe}$$

Accordingly, the air-gap torque, *T_e_*, should better be expressed as:

(17) $$T_{e}=\frac{P_{a}}{{Ω}_{1}}=\frac{1}{{Ω}_{1}}\cdot \left(Re\left\{3\cdot (U-Z_{s}\cdot I_{s})\cdot I_{r}^{\prime *}\right\}\right)=\frac{1}{{Ω}_{1}}\cdot \left(Re\left\{3\cdot (U-Z_{s}\cdot I_{s})\cdot I_{s}^{*}\right\}-P_{fe}\right)$$

Consequently, the following describes the estimation of the air-gap torque, *T_e_*, and the power output, *P_2_*, according to (11) and (12), respectively:

- It only considers the resistive component, *R_s_*, of the stator impedance, ***Z_s_*** = *R_s_* + ***j***·*X_s_*, since the determination of the stator impedance requires the blocked-rotor test, which is not possible in field conditions.
- It only takes into account the stator current, ***I_s_***, since the rotor current, ***I_r_***, (or ***I’_r_***) is unavailable.
- It ignores the magnetic core losses, *P_fe_*.
- To estimate the output power, the method replaces the actual values of the additional load losses, *P_LL_*, and the friction and windage losses, *P_fw_*, with estimates based on the motor rated power according to standards since it is not possible to determine the actual values of these two quantities under field conditions.

**Figure 8:**
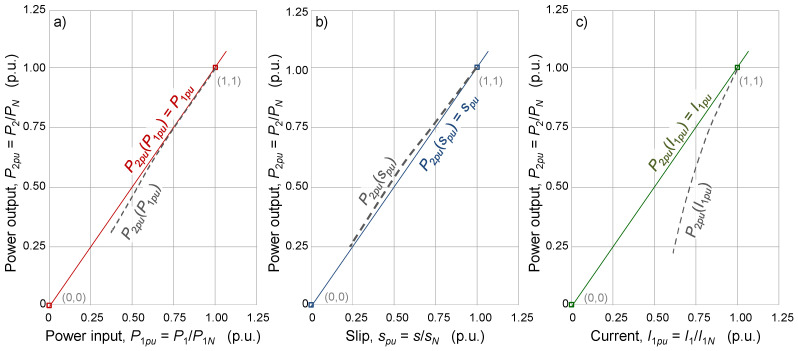
Estimation of the power output of an induction motor according to (**a**) the nameplate, (**b**) slip, and (**c**) current methods (accurate rated nameplate values).

The remaining methods, the nameplate, slip, and current methods, are based on the proportionality between the mechanical power output and the electrical power input, the slip, or the current, respectively, as sketched in Figure 8, assuming that the rated nameplate values were accurate. To make the methods easy to use, the slope of the straight line that approximates the relationship between the power output and the power input, slip, or current is determined without any measurement. The background information (the model) for all these methods is taken from the rated values specified in the nameplate of the motor and, consequently, the slopes of the linear relationships are conditioned by the tolerances allowed by the standards.

Consequently, the estimation of the mechanical power output, *P_2_*, according to (5), (7) or (9) suffers from the following:

- Errors due to estimates being based on linear relationships, where none of them are actually linear (especially the one based on the current).
- Errors due to the tolerances allowed by the standards for the rated values stated on the nameplate.

As can be seen, the “model” of the air-gap torque method, to estimate the output power based on input power and speed measurements, in addition to some approximations, needs to replace some motor data that are necessary but not available, using some kind of generic external information (from standards, for example). Something similar happens with other conventional methods, whose “models” estimate the output power based on the input power, slip, or current measurements, which, in addition to certain approximations, need to use information from the motor nameplate. The specific approximations of each of the conventional methods, the use of generic information in substitution of some necessary but unavailable motor data, or the rated values of the quantities indicated on the motor nameplate (tolerances) contribute to the deterioration of the quality of the estimates.

## 3. Materials and Methods

**Figure 9:**
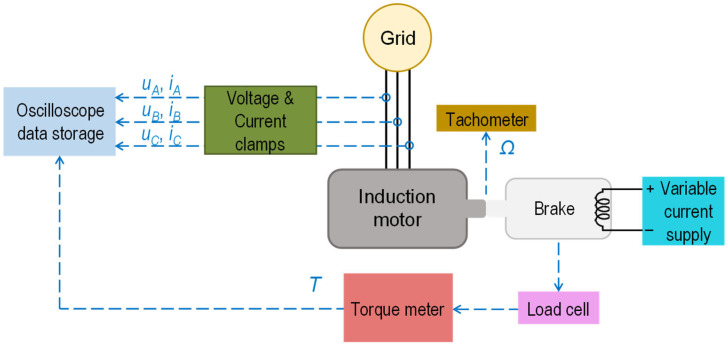
Diagram of the induction motor setup supplied from the grid and coupled to an eddy current dynamometer brake (torque meter).

**Figure 10:**
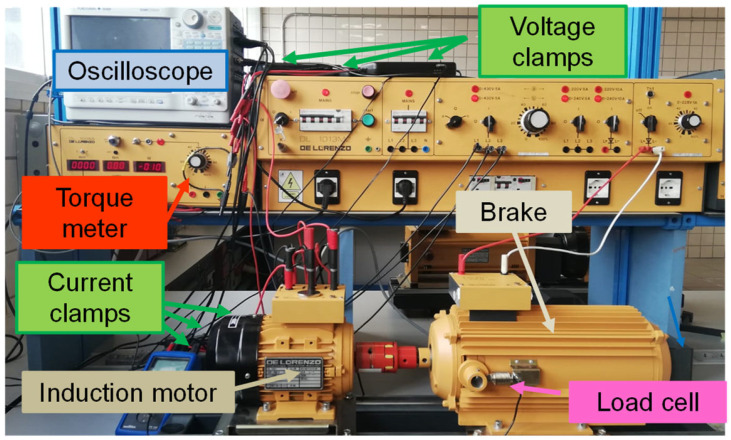
Induction motor layout measurement and test equipment used in the laboratory.

A series of tests were conducted in the laboratory on a 1.1 kW, 2800 r/min, 220 V/380 V, 50 Hz, 4.5 A/2.6 A induction motor with a rated power factor of 0.85, as specified on the nameplate. The motor is installed on a test bench and connected to an eddy current dynamometer brake, which acts as a variable mechanical load and provides torque measurements. By adjusting the brake’s supply, results were obtained for nine operating points ranging from the lowest load condition (denoted as L1) to just above full load (denoted as L9). The motor’s speed was measured using a portable optical tachometer. Appendix A (Table A1, Table A2, Table A3, Table A4, Table A5 and Table A6) includes details on the induction motor and the test equipment used in this work. Figure 9 displays a schematic of the motor setup, and Figure 10 shows the setup in the laboratory.

As shown in Figure 9 and Figure 10, voltage and current clamps are connected to a digital oscilloscope, which is also used as data storage equipment. The output of the torque meter is also connected to the oscilloscope to record the torque values. For each of the tests, thirty-second samples of the instantaneous values of the voltages and currents, as well as the torque, were recorded on the oscilloscope with a sample frequency of 10 kHz.

The power input to the motor, *P_1_*, corresponds to the average value of the instantaneous power over one (Δ*t* = 1/*f*) or several cycles (*N*·Δ*t*) of the grid voltage [38].

(18) $$P_{1}=\frac{\int_{0}^{N\cdot ∆t}\left(v_{A}\cdot i_{A}+v_{B}\cdot i_{B}+v_{C}\cdot i_{C}\right)\cdot dt}{N\cdot ∆t}$$

Table 2 presents the values of the input electrical measurements, the mechanical measurements, and the efficiency of the motor for different partial loads, determined directly using (1).

In the row labeled “*Interp.*”, interpolated values corresponding to full load are added, since the exact values for the point *P*_2_ = 1.1 kW could not be measured. Table 3 shows a comparison between the full-load measurement results of the main quantities and the values printed on the motor’s nameplate, as well as the relative errors compared to the measurements.

As it can be seen, in this case, when comparing the rated values specified on the nameplate with the measured values, it turns out that the absolute errors in power factor, torque, and speed from the nameplate are equal to or less than 0.75%. The absolute errors in the power output, current, power input, and efficiency are, approximately, in the interval 3–4.5%, while the greatest discrepancy occurs in slip and total power losses, with absolute errors from 10% to 17.5%.

The method proposed in this work has two well-differentiated stages: identification and application. In the first stage, measurements of input electrical power and speed are used to identify the coefficients of the estimation model. Once the model has been identified, the second stage allows the output power to be estimated either from speed or input power measurements.

In the first identification stage, the estimation of the mechanical power output, ${\tilde{P}}_{2}$, is carried out by means of the expression:

(19) $${\tilde{P}}_{2}=k_{Ps}\cdot s\cdot (1-s)=k_{Ps}\cdot x_{s}$$

where *k_Ps_* is a proportionality coefficient and the new variable, *x_s_*, is called the slip binomial.

On the other hand, the estimation of total power losses, ${\tilde{P}}_{L}\left(P_{2}\right)$, is conducted using the following quadratic binomial model on the power output, ${\tilde{P}}_{2}$:

(20) $${\tilde{P}}_{L}\left(P_{2}\right)=k_{L0}+k_{L2}\cdot {{\tilde{P}}_{2}}^{2}$$

This loss model is similar to that suggested by the technical specification IEC/TS 60034-31 [30] and is used in [31] for evaluating the energy consumption and life cycle costs of a motor.

After defining the total power loss model as a function of the power output, (20), total losses can also be estimated based on the slip binomial, *x_s_*, as follows:

(21) $${\tilde{P}}_{L}\left(P_{2}\left(x_{s}\right)\right)={k_{L0}+k_{L2}\cdot {\tilde{P}}_{s}^{2}=k}_{L0}+k_{L2}\cdot k_{Ps}^{2}\cdot x_{s}^{2}$$

On the other hand, the estimation of the input power, ${\tilde{P}}_{1}$, can be expressed as the sum of the total losses and the output power:

(22) $${\tilde{P}}_{1}\left(P_{2}\right)={\tilde{P}}_{2}+{\tilde{P}}_{L}\left(P_{2}\right)≃{\tilde{P}}_{2}+k_{L0}+k_{L2}\cdot {\tilde{P}}_{s}^{2}=k_{L0}+{\tilde{P}}_{2}+k_{L2}\cdot {\tilde{P}}_{s}^{2}$$

In summary, the estimations of the output power, ${\tilde{P}}_{2}\left(x_{s}\right)$, total power losses, ${\tilde{P}}_{L}\left(x_{s}\right),$ and input power, ${\tilde{P}}_{1}\left(x_{s}\right),$ can be expressed in terms of the slip binomial, *x_s_*, in the following way:

(23) $${\tilde{P}}_{2}\left(x_{s}\right)=k_{P_{s}}\cdot x_{s}$$

(24) $${\tilde{P}}_{L}\left(x_{s}\right)=k_{L0}+k_{L2}\cdot {\tilde{P}}_{2}^{2}\left(x_{s}\right)=k_{L0}+k_{L2}\cdot k_{P_{s}}^{2}\cdot x_{s}^{2}$$

(25) $${\tilde{P}}_{1}\left(x_{s}\right)={\tilde{P}}_{2}\left(x_{s}\right)+{\tilde{P}}_{L}\left(x_{s}\right)\;\approx \;k_{L0}+{\tilde{P}}_{2}\left(x_{s}\right)+k_{L2}\cdot {\tilde{P}}_{2}^{2}\left(x_{s}\right)\;{\;=k}_{L0}+k_{P_{s}}\cdot x_{s}+k_{L2}\cdot k_{P_{s}}^{2}\cdot x_{s}^{2}=k_{0}+k_{1}\cdot x_{s}+k_{2}\cdot x_{s}^{2}$$

where the new coefficients of the input power polynomial, *k*_0_, *k*_1_, and *k*_2_, can be easily related to the coefficients of the approximations of the output power and total power losses, *k_Ps_*, *k*_*L*0_, and *k*_*L*2_. Thus, the estimation of the electrical power input, (25), turns out to be a second-order polynomial in the variable *x_s_*, depending on slip or motor speed. The three coefficients *k*_0_, *k*_1_, and *k*_2_, appearing in the polynomial ${\tilde{P}}_{1}\left(x_{s}\right)$ can be calculated by setting up a system of equations with three unknowns, using at least one set of measured values of input power, *P_1_*, and slip binomial, *x_s_* (speed), corresponding to three different load points. However, a better way to obtain these coefficients would be to use a wide set of measured points (*x_s_*, *P_1_*), corresponding to a broad range of loads of the motor, and determine the coefficients by minimizing the error in the input power. For this purpose, the following optimization problem is set:

(26) Min∑i=1nP~1,i−P1,i2 subjetc to:       P~1,i=k0+k1·xs,i+k2·xs,i2         ∀ i=1:n xs,i=si·1−si           ∀ i=1:n k0,k1,k2≥0

In this sense, a straightforward option involves deriving these coefficients from the regression polynomial resulting from the set of measured points (*x_s_*, *P*_1_).

Once the values of the three coefficients, *k*_0_, *k*_1_, and *k*_2_, have been determined, the coefficients for the approximations of the output power and total power losses, *k_Ps_*, *k*_*L*0_, and *k*_*L*2_, can be calculated by solving the system:

(27) $$k_{0}=k_{L0}\;k_{1}=k_{Ps}\;k_{2}=k_{L2}\cdot k_{Ps}^{2}$$

Thus, the coefficients for the output power and loss models are obtained as follows:

(28) $$k_{L0}=k_{0}\;k_{Ps}=k_{1}\;k_{L2}=k_{2}/k_{Ps}^{2}$$

Based on the coefficients obtained in (28) and slip binomial (speed) measurements, it is possible to estimate the value of the output power, ${\tilde{P}}_{2}$, via (23) and the efficiency, $\tilde{\eta }$, using (1). This procedure of estimation based on speed (slip binomial) measurements is the most direct way to carry out the second stage of application of the estimation model. However, the polynomial curve (25) that relates input power as a function of the slip binomial (speed), ${\tilde{P}}_{1}(x_{s})$, allows for implementing the second phase of the estimation model based on input power measurements (SCADA) without the need of using speed measurements. For each value of the measured input power, (25) or (26) is used to solve for the corresponding value of the slip binomial, *x_s_*(*P*_1_):

(29) $$x_{s}\left(P_{1}\right)=-\frac{k_{1}}{{2k}_{2}}+\sqrt{{\left(\frac{k_{1}}{{2k}_{2}}\right)}^{2}+\frac{{P_{1}-k}_{0}}{k_{2}}}$$

Once the slip binomial, *x_s_*(*P*_1_), has been calculated, (23) and (1) can be used to estimate the power output, ${\tilde{P}}_{2}\left(x_{s}(P_{1})\right)$, and the efficiency, $\tilde{\eta }\left(x_{s}(P_{1})\right)$, respectively, as previously explained.

**Figure 11:**
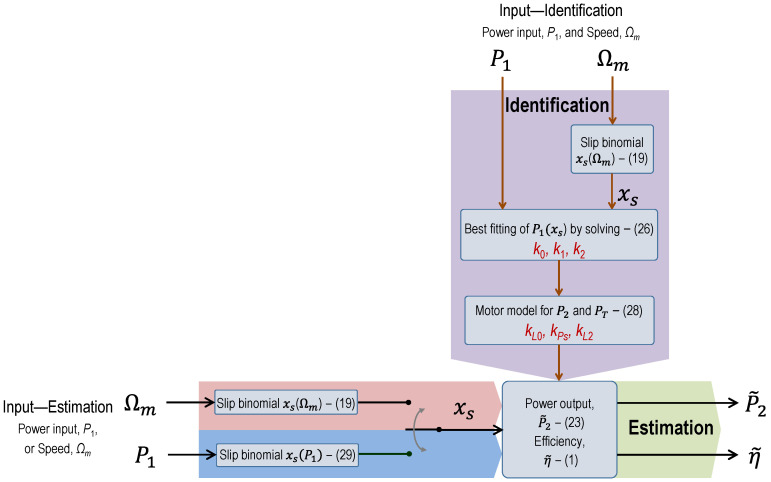
Flowchart of the proposed method for estimating the output power and the efficiency, distinguishing the identification stage (up–down) and the estimation stage (left–right).

The method is outlined in the flowchart provided in Figure 11, which shows the two stages of the proposed method: the identification phase (*P*_1_ and *Ω_m_* measurements) and the estimation phase (*P*_1_ or *Ω_m_* measurements).

Ultimately, the percentage relative error, *ε*, for each load condition is determined as the ratio of the absolute difference between the estimated magnitude, $\tilde{M}$, and the measured value, *M*:

(30) $$\varepsilon =\frac{\left|\tilde{M}-M\right|}{M}=\left|\frac{\tilde{M}}{M}-1\right|$$

and the mean absolute percentage error, MAPE, to assess the overall error of the estimates, ${\tilde{M}}_{i}$, from the set of *N* measurements, $M_{i}$, by means of:

(31) $$MAPE=\frac{1}{N}\cdot \sum_{i=1}^{N}\left|\frac{{\tilde{M}}_{i}-M_{i}}{M_{i}}\right|=\frac{1}{N}\cdot \sum_{i=1}^{N}\left|\frac{{\tilde{M}}_{i}}{M_{i}}-1\right|$$

The standard deviation, *σ*, is used as a measure of the dispersion of the results. It is calculated as:

(32) $$\sigma =\sqrt{\frac{1}{N}\cdot \sum_{i=1}^{N}{\left(M_{i}-M_{AV}\right)}^{2}}$$

where *N* represents the number of measures and *M_AV_* is the mean value of the dataset.

A set of dedicated routines based on MATLAB, version 2022b, were developed to perform the various optimization processes and calculate the estimates of power output, efficiency, and all other quantities shown in Section 4, including the results to support tables and graphs. The MATLAB routines were run on a PC with a 13th Gen Intel(R) Core(TM) i7-1360P 2.60 GHz processor and 32.0 GB RAM, running a 64-bit version of Microsoft Windows 11.

## 4. Results and Discussion

The identification phase of the proposed method is based on the use of a set of measurements, (*x_s_*, *P*_1_), of the input power, *P*_1_, and the slip binomial, *x_s_* = *s*·(1 − *s*). By adjusting the set of input power and motor speed measurements (*x_s_*, *P*_1_) through optimization, expressed in (26) and using (28), the values of the coefficient *k_Ps_*, associated with the output power estimation, and the coefficients *k*_*L*0_ and *k*_*L*2_, associated with the total power losses estimation, are obtained.

**Figure 12:**
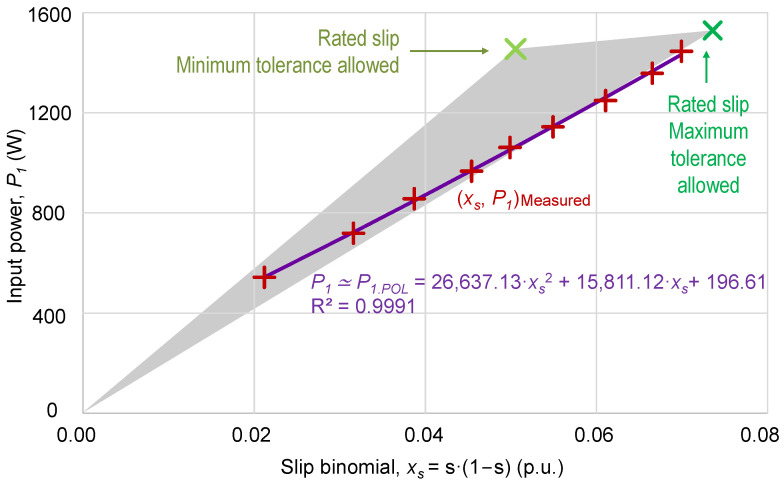
Variation in the input power, *P*_1_, with the slip binomial, *x_s_*, and representation of the experimental points with the corresponding regression curve (second-order polynomial). Gray triangle of indeterminacy due to tolerances.

Figure 12 depicts the set of measured values (*x_s_*, *P*_1_) of the input power and the slip binomial for each partial load. It also shows, through regression, the relationship between the input power, *P*_1_, and the variable, *x_s_*, using a second-degree polynomial, *P*_1,*POL2*_(*x_s_*):

(33) $$P_{1}\left(x_{s}\right)≃P_{1,POL2}\left(x_{s}\right)=196.6141+15,811.1205\cdot x_{s}+26,637.1287\cdot x_{s}^{2}\;R^{2}=0.9991$$

Taking into account the coefficients of the regression curve from (33) and expression (25), each of the terms of the polynomial, i.e., *k*_0_, *k*_1_, and *k*_2_, proposed by this method are identified. Table 4 shows the results.

Once the coefficients of the estimation models have been identified, Table 5 shows the measured values of input power, *P_1_*, and slip binomial, *x_s_*, alongside the calculated slip binomial, *x_s_*(*P_1_*), using (29). Furthermore, the table also displays the measured output power, *P_2_*, the estimated output power derived from speed measurements, ${\tilde{P}}_{2}(x_{s})$, and the estimated output power derived from input power measurements, ${\tilde{P}}_{2}(x_{s}(P_{1}))$. The final columns of Table 5 depict the measured efficiency, *η*, and the estimated efficiency based on speed measurements, $\tilde{\eta }(x_{s})$, and based on input power measurements, $\tilde{\eta }(x_{s}(P_{1}))$, across various partial loads of the motor (L1 to L9).

Table 6 shows the absolute value of the percentage relative error for each estimated variable, *ε_P2_*, and *ε_η_*, corresponding to different speed and input power measurements. Additionally, the average error obtained in the estimation of each variable, *MAPE*_*P*2_ and *MAPE_η_*, are also shown.

As can be seen in Table 6, the values of the relative errors in the estimates of output power, *ε*_*P*2_, and efficiency, *ε_η_*, coincide, because:

(34) $$\varepsilon_{P2}=\left|\frac{{\tilde{P}}_{2}}{P_{2}}-1\right|=\left|\frac{{\tilde{P}}_{2}}{P_{1}}\cdot \frac{P_{1}}{P_{2}}-1\right|=\varepsilon_{\eta }$$

Table 6 shows that in the worst-case scenario, when speed measurements are used, the errors are always below 5.2%. When input power measurements are used, the errors are below 4.8%. Table 6 also shows that the overall errors (MAPE) for the estimations are below 3.5% for output power and efficiency, regardless of the type of measurement employed, whether speed or input power.

### 4.1. Comparison of Results with Other Methods

In this section, the results of the proposed method are compared with those obtained using the conventional methods most used in industry for load and efficiency estimation. These include (i) the nameplate, (ii) the slip, (iii) the current, and (iv) the air-gap torque methods.

Table 7 shows the values of the measured output power, *P_2_*, and the values of the estimates obtained with the nameplate method by (5), ${\tilde{P}}_{2}$^NP^, the slip method by (7), ${\tilde{P}}_{2}$^S^, the current method by (9), ${\tilde{P}}_{2}$^C^, and the air or air-gap torque method by (12), ${\tilde{P}}_{2}$^AGT^. Estimates of the output power derived from speed measurements, ${\tilde{P}}_{2}\left(x_{s}\right),$ and derived from input power measurements, ${\tilde{P}}_{2}(x_{s}(P_{1}))$, obtained by solving (26), are also included.

Table 8 shows the corresponding absolute values of the relative percentage errors for each conventional method and for the proposed method under different load scenarios. The last rows of Table 8 also show the overall errors and the standard deviations for each method. The results of the conventional methods showed that, for the worst-case scenario, the absolute relative percentage error ranges from 89.5% for the current method to 10.6% for the slip method, while for the proposed method, the worst errors are 5.1% (speed) and 4.7% (input power). The values of the global errors for the conventional methods vary from 19.4% for the current method to 4.8% for the nameplate method, while for the proposed method, the global error is reduced to 3.4%.

The standard deviation of the estimates of the conventional methods ranges from 27.27% for the current method to 1.43% for the slip method; this value is reduced to 1.21% (speed) or 1.06% (input power) with the proposed method. The standard deviation of the estimates from conventional methods ranges from 27.27% for the current method to 1.43% for the slip method. The proposed method reduces this value to 1.21% (speed) and 1.06% (input power), suggesting it leads to more consistent and less dispersed estimations.

**Figure 13:**
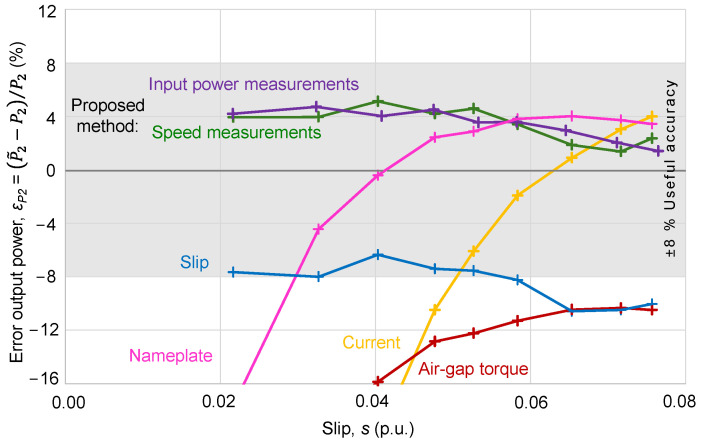
Variation in the error in output power with slip for each conventional method and the proposed method.

Figure 13 shows a graphical comparison of the error in the power output, *ε*_*P*2_, with slip, *s*, for each method, including the limits of “useful accuracy” of ±8%, as introduced by Sousa-Santos et al. in [13], based on the consideration of the steeped values of the rated power commercially available developed by Ferreira and Almeida [16]. As can be seen, only the proposed method keeps all the estimations within the limits of ±8% of “useful accuracy”. In fact, the proposed method goes further, and it is able to keep all estimates within a narrower range of ±5.25%.

**Figure 14:**
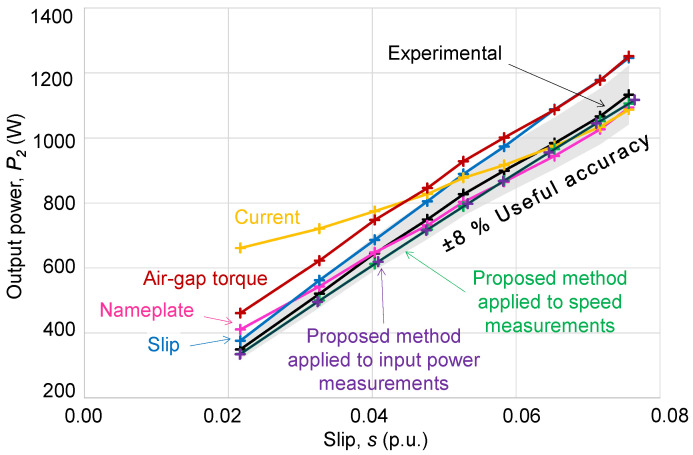
Variation in the output power with slip for conventional methods and for the proposed method.

Figure 14 shows a graphical comparison of the output power variation curves, *P*_2_, with slip, *s*, for each method, including the aforementioned limits of “useful accuracy” of ±8%. This figure, along with the results from Table 8, shows that the errors are greater when estimating the output power using the current method, especially for light loads.

The estimations obtained with the air-gap torque method show an evolution with slip similar to the experimental curve, although this method systematically predicts output power values about 105 W above the actual values.

The slip method also leads to power estimations that are somewhat higher than experimental values, with an increasing difference as the motor load increases.

The nameplate method shows higher errors with low loads when the motor’s efficiency deviates more from its full-load value (hypothesis of the method). It is important to highlight how the permissible tolerances set by standards for the values listed on the nameplate cause the estimation of the nominal output power (1093.6 W) not to match the full-load value (1100 W), although it is close in this specific case. If the nameplate values were correct (hypothesis), the estimated output power curve would shift upward to align with the real curve at the full-load point. At that point, the estimation error would be zero, but errors in the estimations with loads below the rated load would significantly increase (nearly doubling).

Finally, the output power estimation results obtained with the proposed method vary with slip in the same way as the experimental results and, although they slightly underestimate the output power, this method shows a better agreement (overall errors and standard deviations) with the experimental results.

### 4.2. Discussion

Similar to the conventional methods, the “model” of the proposed method, which is used to estimate the power output based on measurements of the slip binomial (speed) or power input, is also based on an approximation. In this case, it is based on a polynomial approximation of the input power with the binomial slip and on a second polynomial to estimate the power output from slip binomial (speed) or power input measurements. Nevertheless, unlike conventional methods, the “model” of the proposed method is identified based on actual measurements of the power input and slip binomial (speed) of the motor under real service conditions.

It is worth noting how the polynomial approximation of the proposed method allows for the identification of the motor “model” without the need to use generic external information to replace the information that is necessary but not available, as occurs with the air-gap torque method. It is also worth noting how the polynomial approximations of the proposed method better accommodate the nonlinearities in the relationship between the output power and the slip binomial than the rigid proportionality relationships imposed by the nameplate, slip, or current methods.

With the aim of analyzing the possible influence on the results of the optimization method, the coefficients *k*_0_, *k*_1_, and *k*_2_, are calculated again, but now by minimizing the errors in the input power using other minimization techniques. For this purpose, the optimization problem (26) was solved using dedicated routines based on the *lsqlin* and *quadprog* MATLAB (version: R2022b) solvers. In fact, the values obtained completely coincide with those previously shown in Table 4.

On the other hand, when a motor is monitored in service (SCADA or MCC), the number of available measurement points could be remarkably high. To analyze how the availability of a higher or lower number of measurements affects the process of identifying and optimizing the coefficients of the approximation polynomials of the proposed method, in addition to the set of nine measurements, the following sets were configured by excluding three of the nine measured points (*x_s_*, *P*_1_) in each of them:

- Set 6.1: six measurements (excluding L2, L5, and L8);
- Set 6.2: six measurements (excluding L1, L5, and L9);
- Set 6.3: six measurements (excluding L3, L5, and L7);
- Set 6.4: six measurements (excluding L2, L6, and L9);
- Set 6.5: six measurements (excluding L1, L4, and L8);
- Set 6.6: six measurements (excluding L2, L4, and L9);
- Set 9: nine measurements.

Table 9 lists the overall error values obtained in the estimation of output power MAPE_P2_(*x_s_*) (speed) and MAPE_P2_(*x_s_*(*P*_1_)) (input power) based on the number of measurements available. Table 9 also shows the overall error values obtained in the estimation of efficiency, MAPE_η_(*x_s_*) (speed), and MAPE_η_(*x_s_*(*P*_1_)) (input power). In each case, the corresponding optimization problem (26) was formulated and solved.

As expected, the results in Table 9 show that the overall errors of the estimates vary between 6.40% and 9.17%, depending on the set of six measures used in the identification stage of the proposed method. It also shows that increasing the number of measurements used in the identification stage from six to nine significantly reduces the overall errors to less than 3.5%.

As can be seen, increasing the number of measurements to identify the proposed model reduces the overall errors in the estimates, which is a distinctive and very favorable feature/characteristic of the proposed method.

Once the expected accuracy and low level of intrusion of the proposed method have been tested, it must be evaluated in terms of other requirements. The equipment required by the proposed method is similar to that of the nameplate or the slip methods. In the worst-case scenario, if input power measurements were not available in the SCADA, it would be like the air-gap torque method. The proposed method does not require any heuristic information (catalogs, standards, or databases), and the data processing requirements would be similar to the air-gap torque method.

The estimation algorithm of the proposed method involves the optimization of a set of coefficients; therefore, at first glance, it does not seem as simple as those used in conventional methods, which (excluding the air-gap torque method) only require the determination of one coefficient. However, this is only partially true since it has been shown that a least squares fit is sufficient to satisfactorily identify the set of coefficients.

Therefore, it can be said that the requirements of the proposed method for what refers to the level of intrusion, instrumentation/equipment, or algorithm/calculation, are quite similar to the conventional methods, while the results outperform those obtained with the classical methods used in industry.

## 5. Conclusions

International standards such as IEC 60034-2-1 [10] and IEEE Standard 112 [11] offer very precise procedures for determining DOL motor efficiency. However, these standards are highly intrusive, requiring equipment like a torque meter and extensive testing, which makes them impractical for field conditions. Consequently, recent research has focused on developing less intrusive methods to estimate motor efficiency. Despite these efforts, a method suitable for field conditions but precise enough to be used with the current I4.0 approach has not yet been established.

To overcome this gap, this work introduces a new kind of non-intrusive and cost-effective method, driven by easily accessible measurements, for estimating the mechanical power output and efficiency of motors in service conditions. The proposed methodology estimates output power using a “slip binomial” approach and approximates total losses with a quadratic binomial model. The method relies on measurements of input power (SCADA) and motor speed (SCADA tachometer), thereby avoiding service interruption.

Despite the level of intrusion, the equipment and complexity of the algorithm are quite similar to conventional methods. The experimental results demonstrate that the proposed method outperforms the conventional methods commonly used in industry, producing more accurate estimates of output power and efficiency. Specifically, the average overall error (MAPE) for output power estimation stands at 3.5%, regardless of whether speed or power input is used in the application or estimation stage; this value is significantly better than the 4.76% error rate of the nameplate method or the 8.48% error of the slip method, which are the best among conventional methods.

Notably, the method shows that increasing the number of measurements to identify the proposed model reduces the overall error of the estimations of the power output and efficiency.

Well-suited for field or in-service conditions, the proposed method requires only readily accessible data, such as input electrical power and speed during the identification stage, and only one of these (input power or speed) during the estimation stage. The proposed method also does not require information on any motor-specific parameters or nameplate data. This adaptability allows the method to be applied to motors that are aged, repaired, rewound, or refurbished, distinguishing it from conventional methods. This also highlights its potential as a practical tool that could help industrial plants achieve the efficiency objectives pursued by the I4.0 philosophy.

Once the utility of the power loss approximation proposed in IEC TS 60034-31:2021 [30] as a basis for estimating output power and motor efficiency has been satisfactorily proven, future work will be focused on the use of more complex approximation functions, cyclic loads, and extending the proposed method to the case of variable speed drives.

## Figures and Tables

**Table 1:** List of tolerances for the values of the quantities declared on the nameplate according to IEC 60034-1:2011 [34].

**Quantity**		**Tolerance**
Rated efficiency, *η_N_*	Rated power, *P_N_* ≤ 150 kW	−15% of (1 − *η_N_*)
	Rated power, *P_N_* > 150 kW	−10% of (1 − *η_N_*)
Rated power factor, *cosφ_N_*		−1.6·(1 − cos *φ_N_*)
		Absolute minimum value: 0.02
		Absolute maximum value: 0.07
Rated slip, s_N_ *	*P_N_* < 1 kW	±30% of *s_N_* *
	*P_N_* ≥ 1 kW	±20% of *s_N_* *

* At full load and operating temperature.

**Table 2:** Results of the measurements of electrical input quantities, mechanical output quantities, and motor efficiency.

Load	Voltage *U* (V)	Current *I* (A)	Power Factor *PF* (p.u.)	Power Input *P*_1_ (W)	Speed *Ω_m_* (r/min)	Torque *T* (N·m)	Slip *s* (p.u.)	Power Output *P*_2_ (W)	Efficiency *η* (%)
L9	381.5	2.56	0.855	1446.1	2773	3.90	0.0757	1132.6	78.32
Interp. *	*380.0*	*2.50*	*0.847*	*1393.2*	*2779*	*3.77*	*0.0736*	*1100.0*	*78.96*
L8	380.4	2.44	0.844	1357.8	2785	3.66	0.0717	1066.5	78.55
L7	381.5	2.30	0.823	1248.6	2804	3.35	0.0653	983.8	78.79
L6	380.4	2.16	0.802	1143.5	2825	3.04	0.0583	899.1	78.63
L5	381.5	2.06	0.778	1061.8	2842	2.78	0.0527	826.8	77.87
L4	380.5	1.95	0.751	966.6	2857	2.50	0.0477	749.3	77.51
L3	380.5	1.83	0.711	856.5	2879	2.14	0.0403	645.1	75.31
L2	381.1	1.70	0.641	718.5	2902	1.71	0.0327	520.3	72.41
L1	380.4	1.56	0.528	542.8	2935	1.14	0.0217	348.9	64.28

* Interpolated values.

**Table 3:** Comparison of the measured values of the full-load quantities with the corresponding values indicated on the nameplate.

Load	Full Load *	Nameplate	Error (%)	Admissible Tolerance
Current, *I* (A)	2.5	2.6	3.85	2.6–3.8
Power factor, *FP* (p.u.)	0.8468	0.8500	0.38	0.61–0.85
Power input, *P*_1_ (W)	1393.2	1454.6 **	4.22	1454.6–1528.4
Speed, *Ω_m_* (r/min)	2779	2800	0.75	2760–2840
Torque, *T* (N·m)	3.7700	3.7515 **	−0.49	3.70–3.81
Slip, *s* (%)	7.36	6.67 **	−10.34	5.34–8.00
Power output, *P*_2_ (W)	1132.6	1100.0	−2.96	–
Total losses, *P_L_* (W)	293.2	354.6 **	17.32	428.6–354.6
Efficiency, *η* (%)	78.9555	75.6232 **	−4.41	71.97–75.62

* Values measured or interpolated from the information in Table 2. ** Value calculated from the nameplate data.

**Table 4:** Results of the coefficients *k*_0_, *k*_1_, and *k_2_* after obtaining the regression curve of the absorbed power against the variable *x_s_*, along with the coefficients *k_Ps_*, *k_L_*_0_, and *k_L_*_2_ associated with the estimates of the output power and total power losses, respectively.

**Coefficients of the Input Power Polynomial**			**Coefficients of the Power Output and Losses Polynomials**		
*k*_0_ (W)	*k*_1_ (W)	*k*_2_ (W)	*k_Ps_* (W)	*k_L_*_0_ (W)	*k_L_*_2_ (W^−1^)
196.6141	1.5811 × 10^4^	2.6637 × 10^4^	1.5811 × 10^4^	196.6141	1.0655 × 10^−4^

**Table 5:** Results of the proposed method: measured and estimated values of slip binomial, output power, and efficiency for each load.

Load	Power Input	Binomial Slip		Power Output			Efficiency		
	*P*_1_ (W)	*x_s_* (p.u)	*x_s_*(*P*_1_) (p.u)	*P*_2_ (W)	${\tilde{P}}_{2}$ (*x_s_*) (W)	${\tilde{P}}_{2}$ (*x_s_*(*P*_1_)) (W)	*η* (%)	$\tilde{\eta }$ (*x_s_*) (%)	$\tilde{\eta }$ (*x_s_*(*P*_1_)) (%)
L9	1446.1	0.0699	0.0706	1132.6	1105.9	1116.6	78.32	76.47	77.22
L8	1357.8	0.0665	0.0661	1066.5	1051.9	1044.9	78.55	77.47	76.95
L7	1248.6	0.0611	0.0604	983.8	965.5	954.9	78.79	77.33	76.47
L6	1143.5	0.0549	0.0548	899.1	868.5	866.9	78.63	75.95	75.81
L5	1061.8	0.0499	0.0504	826.8	788.9	797.5	77.87	74.29	75.10
L4	966.6	0.0454	0.0453	749.3	717.7	715.5	77.51	74.25	74.02
L3	856.5	0.0387	0.0392	645.1	612.0	619.1	75.31	71.45	72.28
L2	718.5	0.0316	0.0314	520.3	499.6	495.7	72.41	69.54	68.99
L1	542.8	0.0212	0.0211	348.9	335.2	334.2	64.28	61.75	61.58

**Table 6:** Results of the proposed method: the absolute value of the percentage relative errors obtained in the estimation of output power, *ε_P_*_2_, and efficiency, *ε_η_*, along with the average error for each variable *MAPE_P_*_2_ and *MAPE_η_*.

Load	*ε_P_*_2_ (*x_s_*) (%)	*ε_P_*_2_ (*x_s_*(*P*_1_)) (%)	*ε_η_* (*x_s_*) (%)	*ε_η_* (*x_s_*(*P*_1_)) (%)
L9	2.36	1.41	2.36	1.41
L8	1.37	2.04	1.37	2.04
L7	1.86	2.95	1.86	2.95
L6	3.40	3.59	3.40	3.59
L5	4.59	3.55	4.59	3.55
L4	4.21	4.51	4.21	4.51
L3	5.13	4.03	5.13	4.03
L2	3.96	4.72	3.96	4.72
L1	3.94	4.20	3.94	4.20
	*MAPE_P_*_2_ (*x_s_*) 3.43%	*MAPE_P_*_2_ (*x_s_*(*P*_1_)) 3.44%	*MAPE_*η*_* (*x_s_*) 3.43%	*MAPE_*η*_* (*x_s_*(*P*_1_)) 3.44%

**Table 7:** Comparison of power output estimates with classical methods and the proposed method as a function of measured values and motor load.

Load	Measured Power Output	Nameplate Method	Slip Method	Current Method	Air-Gap Torque Method	Proposed Method	
	$P_{2}$ (W)	${\tilde{P}}_{2}$^NP^ (W)	${\tilde{P}}_{2}$^S^ (W)	${\tilde{P}}_{2}$^C^ (W)	${\tilde{P}}_{2}$^AGT^ (W)	${\tilde{P}}_{2}(x_{s})$ (W)	${\tilde{P}}_{2}(x_{s}(P_{1}))$
L9	1132.6	1093.6	1246.3	1087.3	1251.3	1105.9	1116.6
L8	1066.5	1026.8	1178.4	1034.2	1176.8	1051.9	1044.9
L7	983.8	944.2	1088.0	975.0	1086.7	965.5	954.9
L6	899.1	864.8	973.2	916.4	1000.7	868.5	866.9
L5	826.8	803.0	889.2	877.1	927.9	788.9	797.5
L4	749.3	731.0	804.7	827.7	845.5	717.7	715.5
L3	645.1	647.7	686.1	774.8	747.4	612.0	619.1
L2	520.3	543.3	561.9	720.8	622.3	499.6	495.7
L1	348.9	410.4	375.6	661.2	460.8	335.2	334.2

**Table 8:** Absolute values of relative errors, average errors, and the standard deviation obtained in the estimation of the output power with the conventional methods and the proposed method.

Load	Nameplate Method	Slip Method	Current Method	Air-Gap Torque Method	Proposed Method	
	*ε_P_*_2_^NP^ (%)	*ε_P_*_2_^S^ (%)	*ε_P_*_2_^C^ (%)	*ε_P_*_2_^AGT^ (%)	*ε_P_*_2_ (*x_s_*) (%)	*ε_P_*_2_ (*x_s_*(*P*_1_)) (%)
L9	3.44	10.04	4.00	10.48	2.36	1.41
L8	3.73	10.48	3.03	10.34	1.37	2.04
L7	4.02	10.59	0.90	10.46	1.86	2.95
L6	3.82	8.24	1.92	11.30	3.40	3.59
L5	2.88	7.54	6.09	12.23	4.59	3.55
L4	2.44	7.41	10.47	12.84	4.21	4.51
L3	0.41	6.35	20.11	15.87	5.13	4.03
L2	4.44	8.00	38.55	19.61	3.96	4.72
L1	17.64	7.66	89.50	32.08	3.94	4.20
	*MAPE_P_*_2_^NP^ 4.76%	*MAPE_P_*_2_^S^ 8.48%	*MAPE_P_*_2_^C^ 19.40%	*MAPE_P_*_2_^AGT^ 15.02%	*MAPE_P_*_2_ (*x_s_*) 3.43*_%_*	*MAPE_P_*_2_ (*x_s_*(*P*_1_)) 3.44%
	*σ_P_*_2_*^NP^* 4.69%	*σ_P_*_2_*^S^* 1.43%	*σ_P_*_2_*^C^* 27.27%	*σ_P_*_2_*^AGT^* 6.69%	*σ_P_*_2_ (*x_s_*) 1.21%	*σ_P_*_2_ (*x_s_*(*P*_1_)) 1.06%

**Table 9:** Results of the proposed method: average overall error values obtained in the estimation of the output power and efficiency based on the number of available measurements.

Number of Measurements	Set of Measurements	MAPE_P2_ (*x_s_*) = MAPE_η_ (*x_s_*) (%)	MAPE_P2_ (*x_s_*(*P*_1_)) = MAPE_η_ (*x_s_*(*P*_1_)) (%)
six measurements	6.1	8.84	8.88
	6.2	6.41	6.40
	6.3	9.11	9.12
	6.4	6.45	6.45
	6.5	8.10	8.12
	6.6	9.17	9.15
nine measurements	9.0	3.43	3.44

## Data Availability

The data presented in this study are available on request.

## Appendix A. Induction Motor and Test Equipment

Table A1, Table A2, Table A3 and Table A4 summarize the main rated characteristics of the induction motor, the dynamometer brake, the torque meter, and the tachometer, respectively, used in this work.

**Table A1:** Main technical characteristics of the induction motor used in the tests.

Motor	Voltage (V)	Current (A)	Power (kW)	Power Factor	Speed (r/min)	Frequency (Hz)
DL 1021 DeLorenzo (2 poles)	220/380 (Δ/Y)	4.5/2.6 (Δ/Y)	1.1	0.85	2800	50

**Table A2:** Technical characteristics of the dynamometer brake used in the tests.

Dynamometer Brake	Maximum DC Supply Voltage (V)	Maximum Speed (r/min)	Maximum Power (kW)
DL 1019 M DeLorenzo	250	4000	1.4

**Table A3:** Technical characteristics of the torque meter used in the tests.

Torque Meter	Measuring Range (r/min)	Accuracy	DC Power Supply
DL 10055 DeLorenzo	0–6000	0.5% full scale	0–220 V, 2 A max

**Table A4:** Technical characteristics of the tachometer used in the tests.

Range	Precision	Number of Digits
2.5 to 99,999 r/min	±0.051% + 1 digit	5

To record voltage and current waveforms, the YOKOGAWA DL850E oscilloscope was used, featuring eight slots for input modules, a sampling rate of up to 100 MS/s, and up to two gigapoints of waveform memory.

Three Fluke i1000s current probes were used to measure current, and three PicoTech (Cambridgeshire, UK) TA042 voltage probes were used to measure voltage. The main characteristics of each are detailed in Table A5 and Table A6.

**Table A5:** Technical characteristics of the current probes.

Current Probe	Range of Rated Current	Range of DC Current	Accuracy	Range of Frequency	Output Ratio
Fluke i1000s	100 A	0.1–100 A	1%	5 Hz–100 kHz	10 mV/A

**Table A6:** Technical characteristics of the voltage probes.

Voltage Probe	Attenuation	Accuracy
PicoTech TA042	1000:1	±2%
